# Loss of Anti-Viral Immunity by Infection with a Virus Encoding a Cross-Reactive Pathogenic Epitope

**DOI:** 10.1371/journal.ppat.1002633

**Published:** 2012-04-19

**Authors:** Alex T. Chen, Markus Cornberg, Stephanie Gras, Carole Guillonneau, Jamie Rossjohn, Andrew Trees, Sebastien Emonet, Juan C. de la Torre, Raymond M. Welsh, Liisa K. Selin

**Affiliations:** 1 Department of Pathology, University of Massachusetts Medical School, Worcester, Massachusetts, United States of America; 2 Department of Biochemistry and Molecular Biology, School of Biomedical Sciences, Monash University, Clayton, Victoria, Australia; 3 Department of Microbiology and Immunology, University of Melbourne, Parkville, Victoria, Australia; 4 Department of Immunology and Microbial Science, The Scripps Research Institute, La Jolla, California, United States of America; Nationwide Children's Hospital, United States of America

## Abstract

T cell cross-reactivity between different strains of the same virus, between different members of the same virus group, and even between unrelated viruses is a common occurrence. We questioned here how an intervening infection with a virus containing a sub-dominant cross-reactive T cell epitope would affect protective immunity to a previously encountered virus. Pichinde virus (PV) and lymphocytic choriomeningitis virus (LCMV) encode subdominant cross-reactive NP_205–212_ CD8 T cell epitopes sharing 6 of 8 amino acids, differing only in the MHC anchoring regions. These pMHC epitopes induce cross-reactive but non-identical T cell receptor (TCR) repertoires, and structural studies showed that the differing anchoring amino acids altered the conformation of the MHC landscape presented to the TCR. PV-immune mice receiving an intervening infection with wild type but not NP205-mutant LCMV developed severe immunopathology in the form of acute fatty necrosis on re-challenge with PV, and this pathology could be predicted by the ratio of NP205-specific to the normally immunodominant PV NP_38–45_ -specific T cells. Thus, cross-reactive epitopes can exert pathogenic properties that compromise protective immunity by impairing more protective T cell responses.

## Author Summary

The purpose of vaccination against viruses is to induce strong neutralizing antibody responses that inactivate viruses on contact and strong T cell responses that attack and kill virus-infected cells. Some viruses, however, like HIV and hepatitis C virus, are only weakly controlled by neutralizing antibody, so T cell immunity is very important for control of these infections. T cells recognize small virus-encoded peptides, called epitopes, presented on the surface of infected cells, and some of these epitopes induce strongly protective and others weakly protective T cell responses. However, the same T cells can sometimes demonstrate cross-reactivity and recognize similar epitopes encoded by two different viruses. We questioned here what infection with a virus encoding a weak cross-reactive epitope would do to immunity to a previously-encountered virus. Here we report that such an infection can compromise protective immunity by enhancing the normally weak response and suppressing the normally strong response. Under these conditions such epitopes function as “pathogenic” epitopes, and we suggest that the potential for inducing responses to pathogenic epitopes should be an important consideration in the design of T cell vaccines.

## Introduction

The desired consequence of vaccination or viral infection is long lasting immunity that protects the host from re-infection or else quickly restricts viral replication to prevent disease and immune pathology. In many cases neutralizing antibody produced by stable plasma cell populations restricts re-infection for the lifetime of the host. In other cases effective neutralizing antibody responses may wane with time or not develop, and resistance relies more on a rapid response by memory T cells [1]. CD8 T cell memory is stable in a pristine environment, but it can be compromised by subsequent viral or bacterial infections [2], [3]. This compromise may be in the form of type 1 interferon (IFN)-induced attrition, resulting in a Bim-dependent apoptosis and loss of memory T cells [4]. Alternatively, this compromise may be in the form of skewing the memory T cell repertoire as a consequence of CD8 T cell cross-reactivity between heterologous agents. Such cross-reactivity is commonplace and is seen in humans between influenza A virus (IAV) and hepatitis C virus (HCV), between IAV and Epstein-Barr virus, and within members of the flavi-, hanta-, and orthomyxo-virus groups [3].

It could therefore be expected that protective immunity could be altered by an intervening viral infection, especially against an agent poorly controlled by neutralizing antibodies and reliant on T cell-dependent immunity, as exemplified by the New World arenavirus Pichinde virus (PV) [5], [6]. PV is distantly related to LCMV, an Old World arenavirus, and these two viruses encode cross-reactive epitopes at nucleoprotein (NP) positions 205–212. Heterologous challenge of LCMV-immune mice with PV results in about a 10-fold reduction in PV titer by day 4 post-infection (PI) when compared to naïve controls, and PV-immune mice synthesize about 2–5 times less LCMV on LCMV challenge [6], [7]. Alterations in the T cell epitope immunodominance hierarchy of the previously immunized animals occurs following heterologous challenge in the LCMV and PV system in either direction [6]. T cell responses to the NP205 epitopes are normally subdominant during infections with either virus alone, even after re-challenge with homologous virus, but in mice sequentially infected with heterologous virus, they become dominant, with narrowly focused oligoclonal repertoires [8].

The beneficial effects of CD8 T cell-mediated clearance of viral infections are sometimes offset by immunopathology, and in experimental models of autoimmunity specific so-called “pathogenic epitopes” may elicit immunopathology due to their cross-reactivity with self-antigens [9]. Herpes simplex virus-1-induced conjunctivitis and Theiler's virus-induced encephalitis are cases where viral epitopes induce cross-reactive T cells that target proteins of the eye and brain, respectively [10], [11]. We questioned here whether select epitopes cross-reactive between two viruses may at times act as pathogenic epitopes and cause immune pathology even in the absence of autoimmunity and show here how an LCMV infection disrupts protective immunity to PV due to the presence of a cross-reactive “pathogenic” epitope.

## Results

### Generation and analysis of NP205 variants

**Figure 1:**
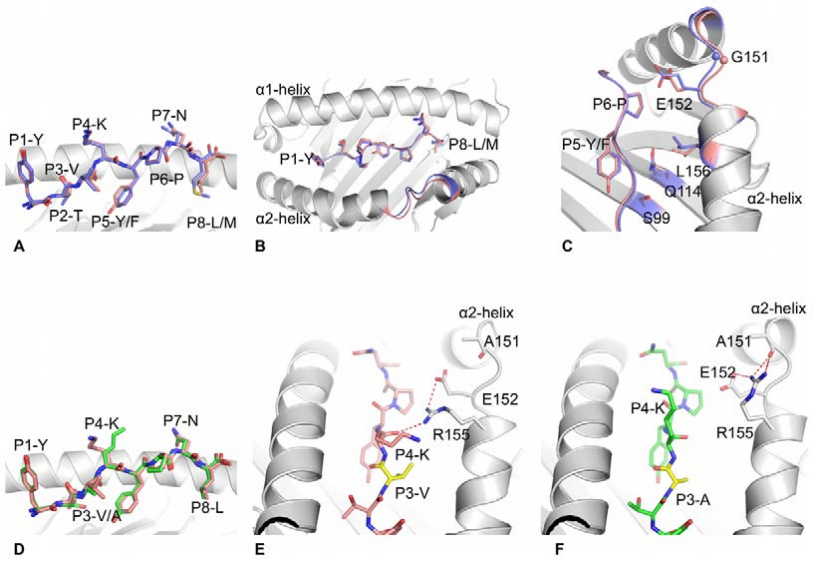
Analysis and comparisons of NP205-K^b^ structures. (A), (B) and (C): superposition of LCMV (pink) with PV (blue) structures, with the peptide in stick representation and the MHC H2K^b^ in grey cartoon. The tip of the α2-helix is colored accordingly to the peptides bound by the H2K^b^ molecules, representing the section from residue 150 to 156 of the α2-helix (B & C). (C) shows, with a different orientation, the residues that change conformation between the peptide-MHC complexes, namely Serine-99, Glutamine-114, Leucine-156, Glutamate-152 as well as Glycine-151, for which the Cα atom is represented by a sphere. (D) superposition of the LCMV (pink) with LCMV-V207A (green) structures, with peptide in stick representation and MHC in grey cartoon. (E) and (F): comparison of LCMV (pink) and LCMV-V207A (green) mutant peptide, both bound to the H2K^b^ molecule (grey cartoon) in the same orientation. The P3 residues are colored in yellow. Arginine-155, Glutamate-152 and Alanine-151 of the H2K^b^ molecule are represented as grey stick to show the different interaction of their side chains between both structures. The red dashed lines represent the hydrogen bond made between the residues.

To analyze the role of cross-reactive epitopes in the elicitation or disruption of protective immunity and immune pathology, we first characterized the molecular properties of wild type and mutant epitopes cross-reactive between LCMV and PV. This study uses both the Armstrong strain of LCMV and its highly disseminating Clone 13 derivative; these viruses differ by only three amino acids and have identical T cell epitopes [12]. LCMV NP_205–212_ (YTVK**Y**PN**L**) and PV NP_205–212_ (YTVK**F**PN**M**) are class I MHC H2K^b^-restricted epitopes that share 6 of 8 amino acids (Figure 1). To evaluate the conformational differences between the LCMV and PV epitopes, we solved the crystal structures of WT H2K^b^ –NP_205–212_ from LCMV and PV to 2.50 Å resolution (Table S1). The structures show that positions 2, 5 and 8 are the anchor residues, whereas positions 2 and 6 are partially exposed, and positions 1, 4 and 7 are solvent-exposed and thus represent potential TCR contact points.

Overall the conformation of the WT LCMV and PV peptides bound to H2K^b^ is similar, with a root mean square deviation (rmsd) of ∼0.24 Å (Figure 1A). The WT NP205 peptides from LCMV and PV differ by only two residues at position 5 and 8, which are MHC anchor residues, and are thus inaccessible for direct TCR contact. The respective H2K^b^ binding clefts adopt similar conformations (rmsd of ∼0.3 Å) (Figure 1B), with the largest difference in a specific region of the α2-helix (rmsd ≃0.5 to 0.9 Å) (Figure 1C). The presence of the P5-Tyrosine hydroxyl group of the LCMV peptide, instead of the P5-Phenylalanine found in the PV peptide, accounts for this perturbation of the α2-helix. Namely, P5-Tyrosine alters the conformation of Serine-99, which is located within the β-strand at the floor of the cleft (Figure 1C), the effect of which is transmitted through the cleft by a rearrangement of side chains of the Glutamine-114, Leucine-156, Glutamate-152 and Glycine-151, for which a maximum displacement of 0.9 Å is observed. This altered positioning of the α2-helix could affect the interaction with the TCR, as differences in this region of the MHC has been shown to impact on TCR ligations in many other systems [13], [14]. This indicates that, although the pMHC complexes are similar, they are not identical epitopes from the perspective of the T cell, and this is reflected by differences in the LCMV-specific vs. PV-specific NP205 repertoires of TCR generated by infection *in vivo* [8].

In our previous study we isolated a T cell escape variant of LCMV Clone 13, where the Valine in the third position of the LCMV NP205 epitope was converted into an Alanine (NP V207A). This mutant epitope stabilized the expression of H2K^b^ on RMA/S cells, indicating that it could be presented by the MHC [8]. The PV-NP205, WT LCMV-NP205, and LCMV V207A mutant peptides had very similar effects at stabilizing H2K^b^ in that the pMHC complexes had an average Tm of 47°C.

To understand the impact of the V207A mutation, the crystal structure of the H2K^b^-NP V207A epitope was determined to 2.30 Å resolution (Table S1). The structure shows that the mutation at P3-Valine of the LCMV peptide into Alanine (NP V207A) did not affect the overall conformation of the H2K^b^ binding cleft (rmsd ≃0.3 Å) (Figure 1D). The difference between the LCMV WT and LCMV-V207A structures is limited to a change in the Arginine-155 conformation between the two pMHC complexes. Namely, within the H2K^b^-NP205 complex, Arginine-155 hydrogen bonds to the main chain of the P4-Lysine residue of the peptide (Figure 1E). In the H2K^b^-NP V207A epitope, on account of subtle movement of the peptide, the conformation of Arginine-155 is shifted such that it now points towards the tip of the α2-helix and hydrogen bonds with the Alanine-151 (Figure 1F). Arginine-155, a position previously termed the gatekeeper residue, has been shown to be involved in interacting with the TCR in most of the structures of TCR-pMHC solved to date and often changes conformation upon TCR ligation [15], [16]. The change of conformation observed for the Arginine-155 due to the Valine to Alanine mutation at position 3 between the LCMV WT and V207A structures explains the effect on the TCR recognition and on T cell activity that is associated with epitope escape.

**Figure 2:**
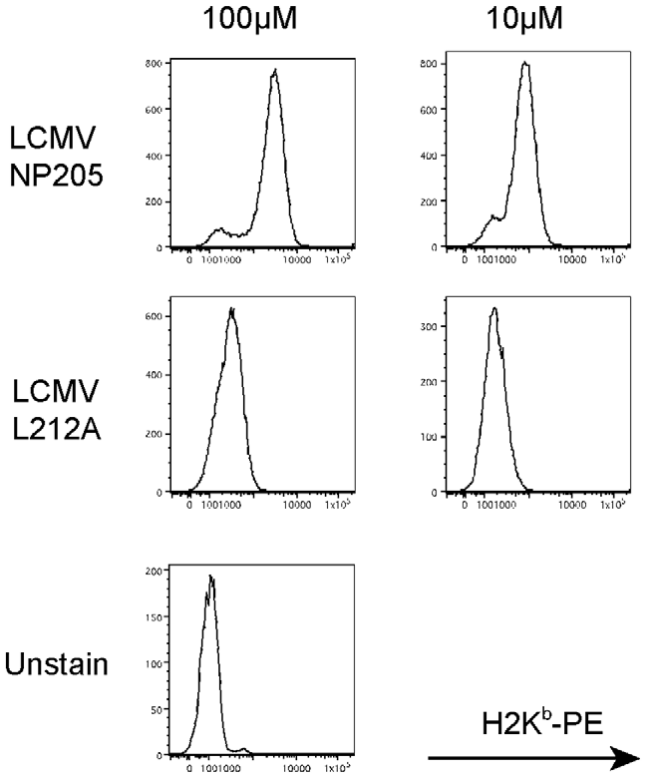
Lack of MHC stabilization by LCMV NP L212A. MHC stabilization assays for LCMV WT (NP205) and mutant (L212A) peptides. RMA-S cells were incubated with different concentrations of peptides and stained against H2K^b^ to detect its stabilization on the cell surface.

Since the naturally selected V207A mutant was generated during LCMV Clone 13 infection and may have had additional mutations, we used reverse genetics approaches to generate rLCMV (rV207A) with the specific mutation V207A within the NP_205–212_ epitope of the Armstrong strain. As a control we also used reverse genetics to rescue WT Armstrong virus (rWT), thereby giving us highly defined viruses differing in a single nucleotide. Because the LCMV-V207A peptides could stabilize H2K^b^ and induce a weak but detectable T cell response, we tested Alanine substitutions in different residues of the NP205 epitope to find a variant that would not stabilize H2K^b^. This was done by converting a Leucine into an Alanine in the eighth (and anchoring) position of the peptide, thereby eliminating MHC stabilization (Figure 2). These results led us to design and generate by reverse genetics the LCMV Armstrong anchoring mutant rL212A. Armed with this assembly of mutant viruses and the knowledge of their structures and biochemical properties we could now address the biological aspects of heterologous immunity between LCMV and PV.

### Reduction in immunogenicity due to point mutations in the LCMV NP205 epitope

**Figure 3:**
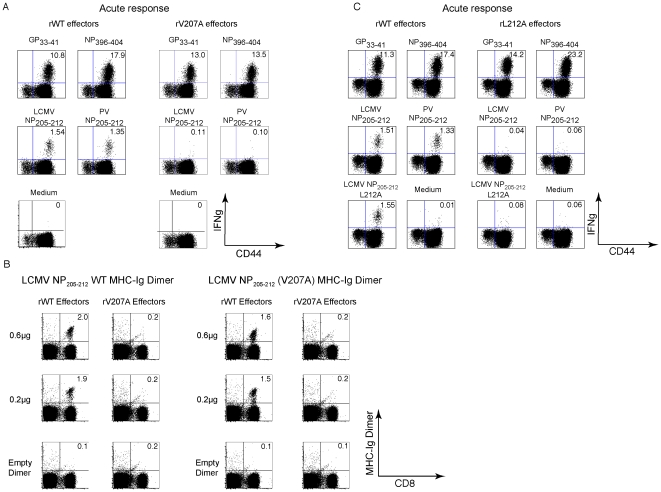
Reduction in immunogenicity as a result of point mutation in the LCMV NP205 epitope (NP V207A). (A) B6 mice (3/group) were inoculated with either rLCMV WT or rV207A variant LCMV-Armstrong. Eight days PI, splenocytes from each group were harvested and stimulated *ex vivo* with a panel of LCMV-specific CD8 T cell peptides for ICS assays. Numbers represent frequencies of IFNγ+, CD8α+ T cells. (B) Splenocytes from rWT- or rV207A-infected mice 8 days PI were stained with LCMV NP205 WT and NP V207A peptide-loaded MHC-Ig dimers. (C) B6 mice (3/group) were inoculated with rLCMV WT or rL212A viruses. Spleens were harvested 8 days PI and stimulated *ex vivo* with indicated peptides. Numbers represent frequencies of IFNγ+, CD8α+ T cells in representative mice.

The newly engineered rV207A variant of LCMV-Armstrong was similar to the natural Clone 13 variant in that it induced normal responses to all tested epitopes except for NP205 (Figures 3A and S1) [8]. Infection with the rV207A LCMV-Arm variant resulted at day 8 PI in greatly diminished responses against either the WT LCMV NP205 or the PV NP205 epitopes (e.g. LCMV NP205 response induced by rWT = 1.8±0.24% vs. rV207A = 0.1±0.05%, n = 3/group, *p* = 0.0002) (Figures 3A and S1). H2K^b^-MHC-Ig dimers were also employed to ensure that the diminished NP205-specific CD8 T cell response in variant-infected mice was due to a loss in specific T cell number and not just due to an alteration in T cell function detected by ICS assays. In the host infected with the rWT virus, similar frequencies of antigen-specific CD8 T cell populations were detected using either LCMV WT NP205-loaded MHC-Ig dimers or LCMV NP V207A-loaded MHC-Ig dimers (2.0±0.1% vs. 1.8±0.25%, respectively, n = 2) (Figures 3B and S1). On the other hand, MHC-Ig dimers loaded with either peptide could detect only a very small percentage of CD8 T cells in mice infected with the rV207A variant virus (Figure 3B). The LCMV Armstrong rL212A anchoring variant, whose NP205 peptide does not stabilize H2K^b^, induced T cell responses well against the LCMV GP33 and NP396 epitopes but failed to induce NP205 responses at all above background (Figures 3C and S1). Note that the L212A peptide did sensitize targets to killing, but that effect was very sensitive to dilution, much as we previously showed with the V207A peptide, in comparison to wild type NP205–212 (data not shown) [8]. These mutants made it possible to assess the role of the NP205 epitope in heterologous immunity.

### Association of cross-reactive NP205-specific CD8 T cells with heterologous immunity

We used the three LCMV mutants that poorly induced NP205-specific CD8 T cell responses to test the hypothesis that heterologous immunity between LCMV and PV was dependent on the NP205 epitope. Naïve controls, LCMV WT immune, and LCMV variant-immune mice were challenged with PV, and PV titers were assessed by plaque assay 4 days PI. PV titers were substantially lower in PV-challenged WT-LCMV-immune mice than in PV-challenged naïve controls (Table 1). These approximately 10-fold reductions in viral titers, while not the sterilizing immunity normally seen during homologous virus challenge, are typical of the reductions seen in heterologous immunity systems and have been shown in other systems to correlate with protective immunity and immunopathology [3]. In contrast, the PV titers in the LCMV NP205 mutant-immune groups were not statistically different from the PV-challenged naïve controls (Table 1). These studies were not done with PV as the first virus and LCMV as the second, because heterologous immunity is weaker in that order of infections, probably due to a lower frequency of NP205-specific memory T cells in PV-immune than in LCMV-immune mice [6]. Nevertheless, these data conclusively show that heterologous immunity can be ablated by a single nucleotide change within a cross-reactive T cell epitope.

**Table 1:** Abrogation of heterologous immunity by point mutation in NP205.

Experiment	Organ	Naïve+PV	WT-immune+PV	Variant-immune+PV
Clone 13 vs. V207A	Spleen	3.6±0.3	2.2±0.7	3.2±0.7
	Fat	3.9±0.4	2.6±0.9	3.5±0.1
rArm vs. rV207A	Spleen	3.7±0.1	2.9±0.2	3.9±0.4
	Fat	4.1±0.2	3.6±0.2	4.5±0.3
rArm vs. rL212A	Spleen	4.3±0.7	3.3±0.3	3.9±0.3
	Fat	4.4±0.2	3.8±0.3	4.5±0.4

Immunologically naïve control or LCMV-immune mice were challenged with 2×10^7^ PFU of PV and tested for PV PFU in spleens or abdominal fat pads 4 days post-infection. Exp. 1 is representative of three experiments using WT LCMV Clone 13 and its naturally derived V207A mutant. Exp. 2 is representative of two experiments using rescued recombinant LCMV Armstrong and its rV207A mutant. Exp. 3 is representative of two experiments using rescued recombinant LCMV Armstrong and its rL212A mutant. n = 5 per group. All comparisons of WT LCMV-immune to naïve mice are *p*<0.05 as indicated by one-way ANOVA analysis and *p*≤0.02 by Students t-test. There was no statistically significant difference in PFU in PV-challenged naïve mice vs. challenged NP205 mutant LCMV-immune mice.

### Acute fatty necrosis (AFN) upon PV re-challenge of double immune mice previously infected sequentially with PV and LCMV

**Figure 4:**
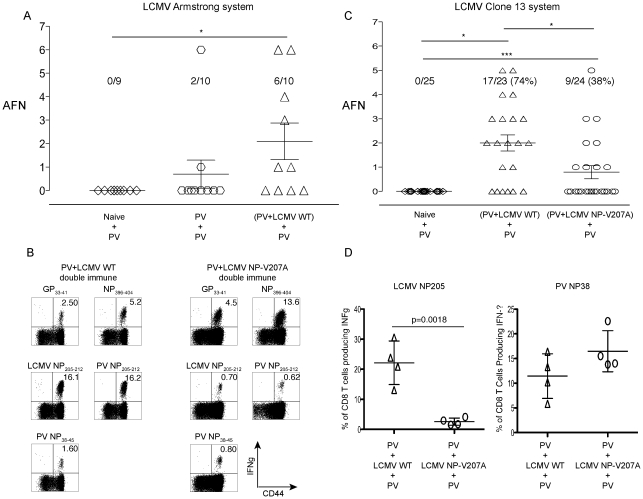
High incidence of AFN in PV+LCMV double immune mice following PV re-challenge. (A) Naïve, PV-immune, and (PV+LCMV WT) double immune mice were re-challenged with PV, sacrificed 3 days PI, and the severity of AFN in the visceral fat pads was assessed. (*) indicates *p*<.05 in frequency of AFN using the Kruskai-Wallis test (one-way ANOVA non-parametric). (B), (C), and (D) represent experiments performed using the LCMV clone 13 system and its naturally derived V207A mutant. (B) Domination of NP205-specific CD8 T cells in PV+Clone 13 LCMV WT double immune mice. PBL were collected from double-immune mice, before the final challenge with PV, and stimulated with peptides *ex vivo* in a standard ICS assay. These are representative frequencies of the IFNγ positive CD8α+ T cells from 4 independent experiments using 5 mice per group. (C) Incidence of AFN after PV challenge. Naïve, (PV+Clone 13 LCMV WT), and (PV+Clone 13 LCMV NP-V207A) double immune mice re-challenged with PV were sacrificed 4 days PI, and the severity of AFN in the visceral fat pads was assessed. Compilation of data from 4 independent experiments. (*) and (***) indicate *p*<.05 and *p*<.0001, respectively. (D) Domination of cross-reactive NP205-specific CD8 T cells isolated from the visceral fat pad of (PV+Clone 13 LCMV WT) double immune mice following PV re-challenge. Standard ICS and FACs analyses were performed. Numbers are representative frequencies of IFNγ+, CD8α+ T cells from two similar experiments.

We next designed experiments to test the hypothesis that an intervening viral infection may disrupt protective T cell-dependent immunity to a previously encountered virus. We chose PV as the first virus, as it does not induce neutralizing antibodies that would interfere with a homologous challenge. Here, PV-immune mice were challenged with LCMV, and these double-immune mice (PV+LCMV) were then re-challenged with PV and assessed for viral titers and immune pathology (Figure 4A). The expectation in this experiment was that the LCMV infection, whether with WT or an NP205 mutant, should reduce the number of immunodominant PV NP38-specific memory cells by IFN-induced attrition [2], [6], [17], as shown by this representative experiment: PV immune only = 5.4±4.2%; PV+rWT LCMV Armstrong = 0.96±0.2%; PV+rV207A LCMV Armstrong = 0.67±0.09%, n = 5/group (*p*<0.05 by Anova test). The next expectation was that the cross-reactive NP205 response, after its initial reduction, should then be amplified in an LCMV-preferred way to form a dominant but narrow oligoclonal response in PV+WT LCMV-immune mice [6], [8]. This LCMV-skewed NP205 response may be less appropriate for effective control of PV.

Our preliminary data with WT viruses surprisingly showed that the double immune mice developed a high incidence and severity of AFN of the abdominal fat pads following the final PV re-challenge (Figure 4A). This AFN was only in rare cases seen in PV-immune mice later re-challenged with PV without the intervening LCMV infection. In some cases (2 of 8 experiments) a loss of protective immunity to PV in regards to virus load was observed upon PV re-challenge of these double immune mice, but most of the time virus could not be detected at day 4 when immune pathology was examined. Clearly, however, rather than there being sufficient protective immunity to prevent disease, the intervening infection disrupted the immunity and predisposed the double-immune mice to an immunopathological disease on re-challenge (Figure 4A).

Knowing that LCMV infection would cause a skewed and oligoclonal expansion of the cross-reactive NP205 epitope-specific T cell pool, we tested for the importance of this epitope, first by using the V207A LCMV Clone 13 variant instead of WT Clone 13 in the viral immunization sequence. The frequencies of antigen-specific CD8 T cells in the blood of the PV+LCMV Clone 13 WT and PV+LCMV Clone 13 V207A double immune mice were monitored prior to the final PV re-challenge. As expected, the cross-reactive NP205-specific CD8 T cell response dominated the immune compartment of the PV+LCMV-Clone 13 WT double immune mice, in contrast to the PV+LCMV-Clone 13 NP-V207A double immune mice (Figures 4B and S1). The average frequency of the cross-reactive NP205-specific CD8 T cells in the PV+LCMV-Clone 13 WT double immune mice before final PV re-challenge was 9.5±6.0% (n = 18) vs. 1.1±0.8% (n = 20) in PV+LCMV-Clone 13 V207A double immune mice (*p* = 0.0019, n = 5/group). These NP205 responses were thus substantially reduced, but, notably, not completely lacking in the mice that received the Clone 13 V207A mutant. As expected, the normally dominant NP38 responses were quite low in the PV+LCMV Clone 13 WT double immune mice (Figures 4B and S1).

The incidence of AFN was higher in the PV+LCMV Clone 13 WT than in the PV+LCMV-Clone 13 V207A double immune mice following PV re-challenge (*p*<0.05 by one way ANOVA non-parametric Kruskai-Wallis test) (Figure 4C). The majority (74%, n = 23) of the PV+LCMV-Clone 13 WT double immune mice displayed AFN as compared to a smaller fraction (38%, n = 25) of the PV+LCMV-Clone 13 V207A double immune mice. In addition, the overall severity of the AFN was higher in the PV+LCMV Clone 13 WT double immune mice re-challenged with PV. These data indicate that a single naturally-derived point mutation in an intervening heterologous virus infection can have a dramatic effect on protective immunity against the first-encountered virus. Although the PV titer in the non-immune naïve group challenged with PV usually reached 10^3^ to 10^4^ PFU/ml in both the spleens and the abdominal fat pads, no AFN was detected at four days PI (n = 25). In these experiments plotted in Figure 4C no PV PFU could be detected in either the spleens or the abdominal fat pads of the PV+LCMV WT and PV+LCMV-V207A double immune mice four days following the PV challenge (n = 23 and 24, respectively). This failure to detect PFU would be a function of the partial immune status of the host and to the relatively late time point at which the organs were harvested.

The frequencies of cross-reactive NP205-specific CD8 T cells in the abdominal fat pads were substantially higher in the PV+LCMV Clone 13 WT than PV+LCMV Clone 13-V207A double immune mice at day 4 following PV re-challenge (22.2±7.6% vs. 2.6±1.4%, *p* = 0.0018, n = 4/group) (Figure 4D). In contrast, the frequencies of the PV NP38-specific CD8 T cells varied less dramatically but trended higher in the PV+LCMV-Clone 13 V207A double immune mice after a PV challenge (11.2±4.4% vs. 16.4±4.3%, respectively, *p* = 0.15). This further implicates a role for the NP205-specific T cells in the immune pathology.

### Complete elimination of immunopathology with the LCMV-Armstrong rL212A anchoring amino acid mutant

**Figure 5:**
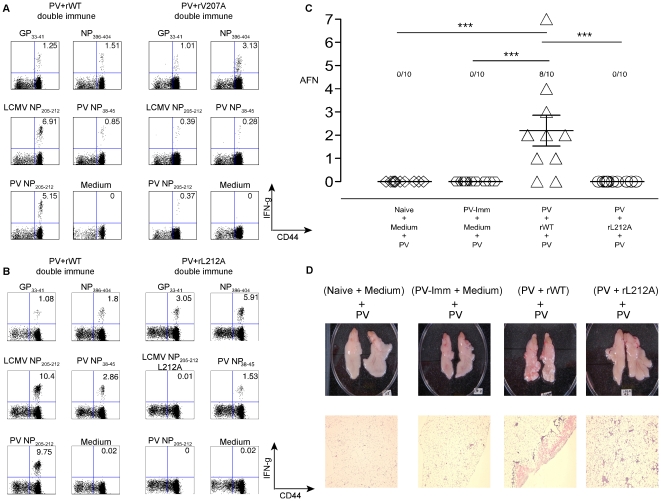
Analysis of immune response and immunopathology with the LCMV-Armstrong rL212A anchoring amino acid mutant. (A) Diminished cross-reactive NP205 CD8 T cell responses in the (PV+rV207A) double immune mice. PV-immune mice were immunized with either rWT or rV207A variant Armstrong strain LCMV. After six weeks, PBL were collected and stimulated with LCMV-specific CD8 T cell peptides. The data represent average frequencies of the IFNγ-positive, CD8α+ T cells. This is representative of 3 experiments, with n = 5/group. (B) Complete elimination of cross-reactive NP205 CD8 T cell responses in (PV+rL212A) double immune mice. PV-immune mice were immunized with either rWT or rL212A LCMV Armstrong. After six weeks, PBL were collected and stimulated with LCMV-specific CD8 T cell peptides. Data represent average frequencies of IFNγ-positive, CD8α+ T cells. This is representative of two experiments, with n = 5/group. (C) Prevention of AFN by the rL212A anchoring mutant. Naïve, PV-immune, (PV+rWT) and (PV+rL212A) double immune groups were re-challenged with PV. Four days later fat pads were harvested and AFN scores evaluated. This is a compilation of two similar experiments. (***) indicates *p*<.0001. (D) Photographs of abdominal fat pads and tissue histology sections. Abdominal fat pads were harvested, photographed (top), and then fixed in 10% neutral buffered formaldehyde and embedded in paraffin at the UMMS histology core facility. Thin tissue sections (5 µm) were stained with hemotoxylin and eosin (bottom). The digital photographs of the sections were taken using a Nikon Eclipse E300 microscope system.

The experiments in Figure 4C were performed over a period of 6 years and used the naturally selected NP V207A mutant in the LCMV Clone 13 system. While this variant elicited markedly reduced NP205-specific responses, the responses were not completely absent in the double immune mice, as shown in Figures 4B and S1, and it was unclear whether the small fraction of NP205-specific T cells induced may have affected the results. We initiated tests with the LCMV-Armstrong rV207A variant (Figures 5A and S1) and found that, as with the natural Clone 13 NP-V207A variant (Figures 4B and S1), there was a reduced but still detectable NP205 response in the double immune mice prior to PV re-challenge. Rather than continuing to explore that variant in extensive pathogenesis studies, we focused on the LCMV-Armstrong rL212A anchoring variant. Figures 3C and S1 show that mice inoculated with the LCMV-Armstrong rL212A mutant generated relatively normal acute T cell responses to the immunodominant LCMV epitopes GP33 and NP396, but there was virtually no response against either the LCMV or PV NP205 peptides or even to the L212A peptide (Figures 3C and S1). Importantly, there also were no NP205-specific memory responses in double-immune mice first immunized against PV and later challenged with the LCMV-Armstrong rL212A variant (Figures 5B and S1).

We next questioned how LCMV-Armstrong rL212A influenced immunopathology in double-immune (PV+LCMV) mice re-challenged with PV. Whereas detectable AFN was found in 80% of the PV+LCMV-Armstrong rWT-immune mice after PV re-challenge, none of the mice in the PV+rL212A-immune group developed AFN. Examples of gross pathology and H&E sections are displayed in Figure 5D. The AFN presented as chalky white areas on the surface of the fat tissue (top) and as pink areas of dying cells in the H&E sections (bottom). These studies with this anchoring-deficient LCMV-Armstrong rL212A mutant strengthen the argument that an intervening heterologous virus infection bearing a cross-reactive epitope can alter immune pathology developing in response to a previously encountered pathogen and that a single base change can abrogate this effect.

### Prediction of the development of immune pathology

**Figure 6:**
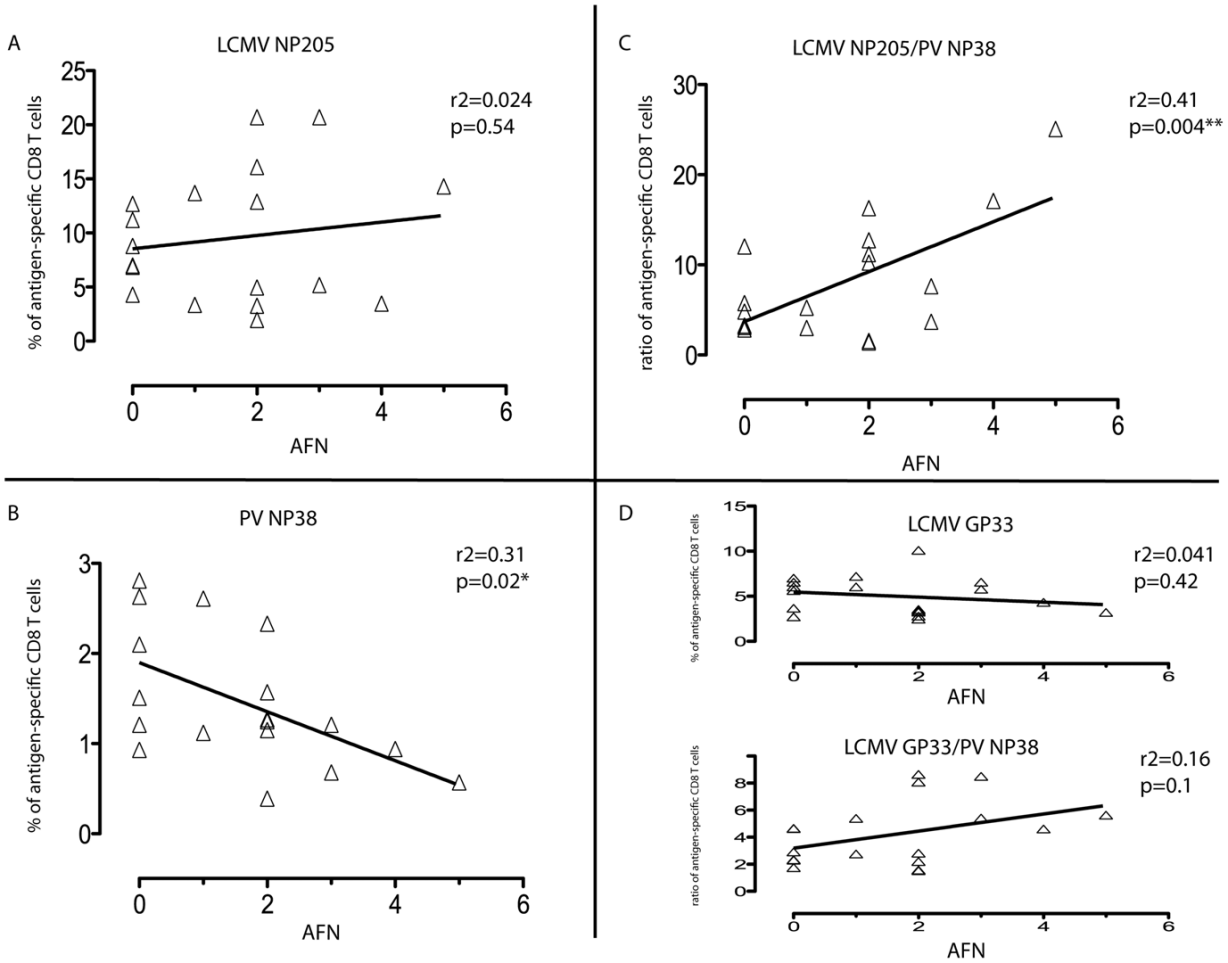
Correlation of frequencies of CD8 T cells in double-immune mice with pathology after PV re-challenge. Linear regression analyses comparing the frequencies of antigen-specific CD8 T cells in the (PV+WT LCMV) double immune mice with the severity of AFN following PV re-challenge. These represent data compiled from four independent experiments using LCMV Clone 13 virus. (A) LCMV NP205-specific CD8 T cell response. (B) PV NP38-specific CD8 T cell response. (C) Ratio of LCMV NP205 to PV NP38. (D) LCMV GP33 and the ratio of GP33/PV NP38.

We next asked if one could predict whether a double-immune host would develop immune pathology on re-challenge, by applying Pearson correlation and linear regression analyses comparing the frequencies of epitope-specific T cells in the PBL of PV+WT LCMV Clone 13 double immune mice prior to PV re-challenge to the degree of the immunopathology seen later on PV re-challenge. There was surprisingly no correlation between the frequency of NP205-specific CD8 T cells in double-immune mice before the PV re-challenge and the severity of the AFN four days later (Figure 6A), but there was a strong negative correlation between the frequencies of the normally immunodominant PV NP38-specific CD8 T cells in the double-immune mice with the severity of the AFN after challenge with PV (*p* = 0.02, n = 18) (Figure 6B). Interestingly, an even more and highly significant positive correlation (*p* = 0.004, n = 18) was seen if the ratio between the cross-reactive NP205-specific CD8 T cells and the PV NP38-specific CD8 T cells was plotted against the severity of AFN (Figure 6C). T cells specific to these epitopes compete with each other [6], and this ratio would likely portend how quickly a protective NP38-specific T cell response could be generated while in competition with the NP205-specific T cells present in higher frequencies. No significant correlation was found between the frequencies of the LCMV GP33-specific CD8 T cells or the LCMV GP33/PV NP38 ratio and the level of AFN (Figure 6D). On a smaller scale with double-immune mice using the rWT Armstrong virus, two experiments that had strong AFN on re-challenge with PV showed positive correlations with the frequencies both of NP205-specific T cells (R^2^ = 0.48; *p* = 0.027) and with the ratio of NP205- to NP38-specific T cells (R^2^ = 0.41; *p* = 0.046) with the severity of AFN (n = 10). Thus, there was predictive value in knowing the frequencies of the cross-reactive and immunodominant PV-specific epitopes.

## Discussion

This report shows that protective immunity to a virus can be disrupted by an otherwise well-tolerated and controlled infection with a second and different virus. Further, it shows that a single cross-reactive CD8 T cell epitope on that second virus can dictate the degree of immune pathology on re-challenge with the first virus. The NP205 epitopes encoded by LCMV and PV are highly cross-reactive because they differ only in their MHC-anchoring amino acids, and our studies presented in Table 1 with NP205 mutants clearly implicate this cross-reactive epitope in protective heterologous immunity between these viruses. However, these epitopes induce distinct TCR repertoires, and sequential infections with these viruses result in very narrowly focused repertoires skewed in favor of the second-encountered virus [8]. These inappropriate T cell repertoires may interfere with strong protective immunity to the first encountered virus.

The effects of buried MHC polymorphisms on TCR recognition have been previously evaluated [8], [18], [19], and we show here that epitope-anchoring amino acids buried within the MHC can alter the conformations of determinants accessible to the TCR [20], explaining why different TCR repertoires can react with these LCMV and PV NP205 epitopes (Figure 1A,B,C). Further, we show how a mutation in the third position of the LCMV NP205 epitope will allow for epitope binding to the MHC yet alter its interaction with T cells generated in response to the wild type epitope (Figure 1D,E,F). Strikingly, single nucleotide changes altering the cross-reactive epitope of the second intervening virus removed its ability to interfere with the protection from disease (Figures 4 and 5). In this case we suggest that the loss of T cells specific to a protective and normally immunodominant epitope (NP38) by a combination of IFN-induced attrition and competition with T cells responding to a normally subdominant cross-reactive epitope (NP205) tips the balance from efficient protective immunity to less efficient immunopathology.

Previous studies, as well as results presented here, have shown that the immunodominant PV NP38 response is substantially reduced in double (PV+LCMV)-immune mice in comparison to PV only-immune mice [6], [17]. Protective T cell-dependent immunity to tumors can be lost after bacterial infections [21], and a recent report shows that protective immunity to *Plasmodium* is lost in mice subjected to a series of infections [22]. In our present study, however, the reduction of the immunodominant NP38-specific T cell response caused by the intervening LCMV infection was partially compensated for by the cross-reactive NP205 response, which became dominant in double immune mice. The price for the increased cross-reactive response, which was not ideal for protection against PV, was enhanced disease associated with immune pathology on PV rechallenge. If LCMV NP205 was mutated in a way (V207A) that resulted in a reduced though still detectable T cell response, the PV+LCMV-double immune hosts responded to PV re-challenge with less pathology; if the intervening LCMV was mutated in an MHC anchoring site (L212A) to prevent any T cell response at all, the PV+LCMV double-immune hosts responded to PV re-challenge with even less pathology, which was undetectable.

The ratio of NP205-specific to NP38-specific T cells in double-immune mice had strong predictive value for the production of immune pathology on re-challenge with PV (Figure 6). The ratios of these epitope-specific T cells in double immune mice might predict their relative abilities to compete with each other in their early response to the PV re-challenge. Analyses of T cells in diseased tissue day 4 after PV re-challenge are complicated by the severe necrosis and collateral cell damage in the adipose tissue, but many NP205-specific T cells are found at that time (Figure 4D). It is likely, however, that T cell responses occurring very early after challenge may have controlled viral load and affected the outcome.

Severe immune pathologies associated with cross-reactive T cell responses in humans have been reported in fulminant HCV-associated hepatitis, infectious mononucleosis, and dengue hemorrhagic fever and shock syndrome [23]–[26]. Aberrant pathology associated with cross-reactive pathogenic epitopes is thus an issue that should be considered in vaccine construction. For instance, some strains of HCV encode an epitope that strongly cross-reacts with an epitope of IAV [27], and HCV vaccines containing this cross-reactive epitope are under evaluation [28]. One wonders what a sequence of an IAV infection (or vaccine) and an HCV vaccination, in either order, would have on a subsequent encounter with either virus. We suggest that these concerns would be less for viruses or viral vaccines that would induce high levels of neutralizing antibody, which might prevent infection in the first place. However, viruses like HCV, HIV, and CMV are relatively poor at inducing effective neutralizing antibody responses, and individuals infected with these viruses often become super-infected with slightly different variants. Cross-reactive pathogenic epitopes might also be an issue with influenza virus infections when individuals with poor neutralizing antibodies but strong cross-reactive T cells to new influenza virus strains become infected.

The panniculitis described in our current model may seem unusual, but panniculitis is a pathology commonly found in humans in the form of erythema nodosum, which involves inflammation of subcutaneous fat tissue [29]. Erythema nodosum sometimes occurs following infections or in association with autoimmune diseases such as Crohn's [30]. Of relevance to our present work, panniculitis is sometimes found in humans after vaccinations for smallpox, hepatitis B and papilloma viruses [31]–[33]. In mice, panniculitis and AFN of visceral fat pads is a common feature of virus infections by the intraperitoneal route, but it is particularly noticeable in models of heterologous immunity, where memory T cells induced by an earlier heterologous viral infection rapidly respond to but inefficiently clear an infection of the fat pads by a second virus [3], [7], [34]. The Armstrong strain of LCMV replicates poorly in the fat pads and does not directly elicit AFN, but a history of an LCMV infection can prime a mouse for AFN after infection with certain heterologous viruses that do grow in the fat. The mechanism of AFN is best studied in LCMV-immune mice infected with vaccinia virus, where cross-reactive T cells enter the fat pads and stimulate necrosis through an IFNγ, TNF, and Fas ligand-dependent mechanism that reflects the private specificity of the T cell populations in LCMV-immune mice, as shown in assays using adoptive transfers of immune T cell [7], [35], [36]. PV, used in the current study, does replicate in fat tissue, and the disruption of the memory T cell response specific to PV by the LCMV infection has apparently created the conditions that predispose to AFN rather than pathology-free clearance of virus on re-challenge with PV.

The unique finding of our current study, however, is not simply another demonstration of heterologous immunity. Rather, it is the finding that the heterologous immunity associated with an intervening infection with a virus containing a cross-reactive epitope can have a profound impact on the homologous immunity against a previously immunized pathogen. Hence, lasting immunity to a previously encountered pathogen can be compromised by subsequent infections with other pathogens bearing cross-reactive pathogenic epitopes.

## Materials and Methods

### Ethics statement

This study was carried out in strict accordance with the recommendations in the Guide for the Care and Use of Laboratory Animals of the U. S. National Institutes of Health. All animal work was reviewed and approved by the UMMS institutional Animal Care Committee (Animal Welfare Assurance # A3306-01), and all the efforts were made to minimize suffering of mice.

### Mice

C57BL/6 (B6, H2K^b^) male mice were purchased from the Jackson Laboratory (Bar Harbor, ME) and maintained under specific pathogen-free conditions at the University of Massachusetts Medical School (UMMS) Department of Animal Medicine. All animal work was reviewed and approved by the UMMS Institutional Animal Care Committee.

### Viruses

The AN3739 strain of PV and several strains and variants of LCMV were propagated in BHK-21 cells. These include LCMV, strain Armstrong, and recombinant (r) Armstrong variants harboring laboratory-directed mutations in the NP_205–212_ epitope: rLCMV wild type (rWT), rV207A, and rL212A. The Clone 13 natural variant of LCMV, which has mutations in the glycoprotein and polymerase that allow for greater replication and dissemination *in vivo* [37] was also used, as well as a naturally-derived CD8 T cell escape variant in the NP_205–212_ epitope, LCMV-clone 13 V207A. Clone 13 can be used at high doses to establish persistent infections, but the experiments described here use lower doses that generate immune responses that clear infection similarly to that of the Armstrong strain. To avoid immune responses generated to bovine serum following sequential infections, PV was purified by sucrose density gradient ultra-centrifugation and diluted in serum-free HBSS before immunization [7]. LCMV stocks were propagated to titers over 10^7^ PFU/ml as assayed on vero cell monolayers and diluted in serum-free HBSS prior to infection of mice.

Experimental procedures used for the generation and rescue of recombinant LCM viruses (rLCMV) were as described [38]. Briefly, BHK-21 cells were transfected with T7 RNA polymerase (T7RNP)-based expression plasmids that directed intracellular synthesis of full-length S and L genome RNA species of LCMV Armstrong strain, together with pol II-based expression plasmids expressing T7RNP and the minimal viral trans-acting factors (L and NP) required for virus RNA replication and gene transcription. At 60 h post-transfection, tissue culture supernatants were collected (referred to as P0), clarified at low speed and used to infect fresh monolayers of BHK-21 cells. At 48 h p.i., TCS were collected (P1) and titrated by plaque assay.

All inoculations were by the intraperitonal route. For primary infections with LCMV, male mice 6–8 weeks of age were inoculated with 5×10^4^ to 5×10^5^ PFU of LCMV. For primary infections with PV, mice were inoculated with 2×10^7^ PFU of purified PV. Mice were considered immune 6 weeks after immunization. For homologous challenge, PV-immune mice were inoculated with 2×10^7^ PFU of purified PV. For heterologous challenge, LCMV-immune mice were inoculated with 2×10^7^ PFU of purified PV. For generation of double immune mice, PV-immune animals were challenged with 5×10^5^ to 1×10^6^ PFU of LCMV WT or LCMV variants. Re-challenge of (PV+LCMV) double immune mice was done with 2×10^7^ PFU of purified PV.

### Synthetic peptides

LCMV-encoded peptide epitopes used were GP_33–41_ (KAVYNFATC), NP_396–404_ (FQPQNGQFI), LCMV NP_205–212_ (YTVKYPNL), mutated NP_205–212_ V207A (YT**A**KYPNL) and L212A (YTVKYPN**A**). PV-encoded epitopes were NP_38–45_ (SALDFHKV) and PV NP_205–212_ (YTVK**F**PN**M**). Synthetic peptides were from BioSource International or 21^st^ Century Biochemicals at 90% purity.

### Peptide/MHC stabilization assay

TAP-1 deficient RMA-S cells [39] were seeded into 96-well U-bottom plates at 5×10^5^ cells per well. Following incubation in 5% CO_2_ at 27°C for 4 hours, variants of LCMV NP205 peptides were added at different concentrations and incubated overnight. The cells were then stained with mAb to H2K^b^ (clone AF6 88.5) conjugated with PE (BD Bioscience) and analyzed by fluorescence-activated cell sorting (FACS).

### Protein expression, purification, crystallization and structure determination

H2K^b^ and β2-microglobulin molecules were expressed in *Escherichia coli* as inclusion bodies, refolded with the LCMV-NP205, PV-NP205 or LCMV-V3A (V207A) NP205 peptides and purified as previously described [40]. The three plasmid (p) MHC complexes were concentrated to 2–5 mg/ml, using the hanging-drop vapor diffusion technique at 20°C. Crystals were grown with a reservoir containing 16–24% polyethylene glycol (PEG) 3350, 0.1 M Na-Cacodylate, pH 6.5, and 0.2 M Na acetate. The crystals belong to space group ***P2_1_*** and the unit cell dimensions were consistent with two molecules per asymmetric units (Table S1).

The crystals were flash frozen to a temperature of 100 K before data collection using an in-house X-ray generator with a RAXIS-IV detector for the H2K^b^-LCMV NP205 or at the Australian Synchrotron on the BM1 beamline with a MarCCD or an ADSC Q210r detector for the H2K^b^-LCMV-V207A (V3A) and H2-K^b^-PV NP205 structures. The data were processed and scaled with the XDS [41]. The crystal structure was solved using the molecular replacement method in the program Phaser [42] from the CCP4 suite of programs (1994). The search probe used to solve the structure was the structure of mouse MHC class I H2K^b^ minus the peptide (Protein Data Bank accession number 2ZSV) [43]. The progress of refinement was monitored by the R_free_ value with neither a sigma nor a low-resolution cut-off being applied to the data. This protocol includes several cycles of refinement with the PHENIX software [44] followed by manual model rebuilding with Coot program [45]. Final refinement statistics are summarized in Table S1. The coordinates of the three complexes have been deposited with the Protein Data Bank under accession numbers **3P4M**, **3P4N** and **3P4O** for the H2K^b^-NP205-LCMV, H2K^b^-NP205-PV and H2K^b^-LCMV-V3A (NP V207A), respectively.

### Thermostability measurements of recombinant class I complexes using circular dichroism (CD)

Circular Dichroism Spectra were measured on a Jasco 815 spectropolarimeter using a thermostatically controlled cuvette. A far-UV spectra was collected from 190 nm to 250 nm. The UV minimum was determined as 219 nm for the three peptide-MHX complexes. The measurements for the thermal melting experiments were made at the minimum, at intervals of 0.1°C at a rate of 1°C/min from 20°C to 90°C. The Jasco Spectra Manager software was used to view and smooth the traces, and then the GraphPad Prism software was used to plot temperature versus % unfolded. The midpoint of thermal denaturation (Tm) for each protein was determined as the point at which 50% unfolding was achieved. The measurements were done in duplicate at two concentrations (5 µM and 10 µM) in a solution of 10 mM Tris pH 8, 150 mM NaCl.

### Acute fatty necrosis (AFN) scores

The severity of AFN was scored based on the guidelines from a previous publication: **(1–2)** very mild to mild disease with a few white necrotic spots on one or both lower abdominal fat pads; **(3–4)** mildly moderate and moderate with larger patches of necrosis of the lower abdominal fat pads and extension into the upper left quadrant fat pad around the spleen; **(5–6)** moderately severe to severe with very extensive large patches of necrosis on the lower abdominal fat pads and spotty fatty necrosis throughout omental fat pads as well as the splenic fat pad; **(7)** very severe disease with such severe fatty necrosis that the organs are adherent to each other [7], [35].

### Tissue histology

Abdominal fat pads from different groups of mice were harvested and fixed in 10% neutral buffered formaldehyde and embedded in paraffin at the UMMS histology core facility. Thin tissue sections (5 µm) were stained with hemotoxylin and eosin. The digital photographs of the sections were taken using the Nikon Eclipse E300 microscope system at the UMMS core facility.

### Isolation of lymphocytes from adipose tissue

The infiltrating leukocytes in the fat pads were isolated by mincing and digesting with collagenase B (200 mg/ml) in MEM plus 4% BSA for 1 hour at 37°C, and then by separation over Lympholyte-M from Cederlane Laboratory (Burlington, ON).

### Intracellular cytokine staining (ICS)

Leukocytes from spleens, blood and abdominal fat pads (1×10^6^ cells/well) were stimulated with 1 µM peptides in medium containing 0.2 µl of GolgiPlug and human recombinant IL-2 (BD Pharmingen) at 37°C for 5 hours. Intracellular cytokine staining (ICS) was performed using a cytofix/cytoperm kit from BD Bioscience. Intracellular cytokine-producing cells were detected with allophycocyanin (APC)-conjugated anti-mouse IFNγ monoclonal antibodies (1∶1000) (XMG1.2) and phycoerythrin-Cy7 (PE-Cy7)-conjugated anti-mouse TNFα (1∶200) (MP6-XT22).

### Cell surface and MHC-Ig dimer staining by flow cytometry

All the surface antibodies were used at a 1∶200 dilution per well. Single-cell suspensions of splenocytes or blood lymphocytes were first incubated with anti-mouse CD16/CD32 Fc-block antibody (1 µl/well) for 15 minutes on ice. Subsequently a cell surface staining procedure was performed using PerCP-Cy5.5 anti-mouse CD8α (clone 53-6.7) and FITC anti-mouse CD44 (clone IM7). The samples were incubated on ice for 20 minutes.

Soluble dimeric mouse H2K^b^-Ig fusion proteins (MHC-Ig dimer) were purchased from BD Bioscience (San Diego, CA). The LCMV WT NP205 and mutant V207A peptides (1 mg/ml) were incubated with the MHC-Ig dimer at an 800 to 1 molar ratio and with recombinant human beta-2 micro-globulin (0.15 µg/µg of dimer) from BD Bioscience at 4°C for 4 days for passive loading of the peptide onto the MHC. The final products were used for surface staining assays as above and previously reported [2].

### Statistical analysis

Statistical analysis was performed using GraphPad Prism software (5.0b). Comparisons between two groups were performed using the unpaired Student's *t* test (2-tailed). Comparisons between more than two groups were performed using one way Anova analysis (2-tailed). Pearson's correlation and linear regression tests were used to measure the correlation between two independent variables. *P* values less than 0.05 were considered statistically significant.

## Supporting Information

Figure S1 **Graphic analysis of dot plots from** **Figures 3** **,** **4** **, and** **5** **.** This figure graphs the magnitude and variance of epitope-specific T cell responses from replicas associated with the representative data presented in Fig. 3A (n = 3/group), 3B (n = 2/group), 3C (n = 3/group), 4B (n = 5/group), 5A (n = 5/group), and 5B (n = 5/group). All show means ± standard deviations (*p*<0.05*).
(TIF)

Table S1 Data collection and refinement statistics.
(DOC)
