# Supplementary figures and images for: Loss of Anti-Viral Immunity by Infection with a Virus Encoding a Cross-Reactive Pathogenic Epitope
Source: PLoS Pathog. 2012 Apr 19;8(4):e1002633. doi: 10.1371/journal.ppat.1002633 (PMC3334890; doi:10.1371/journal.ppat.1002633)

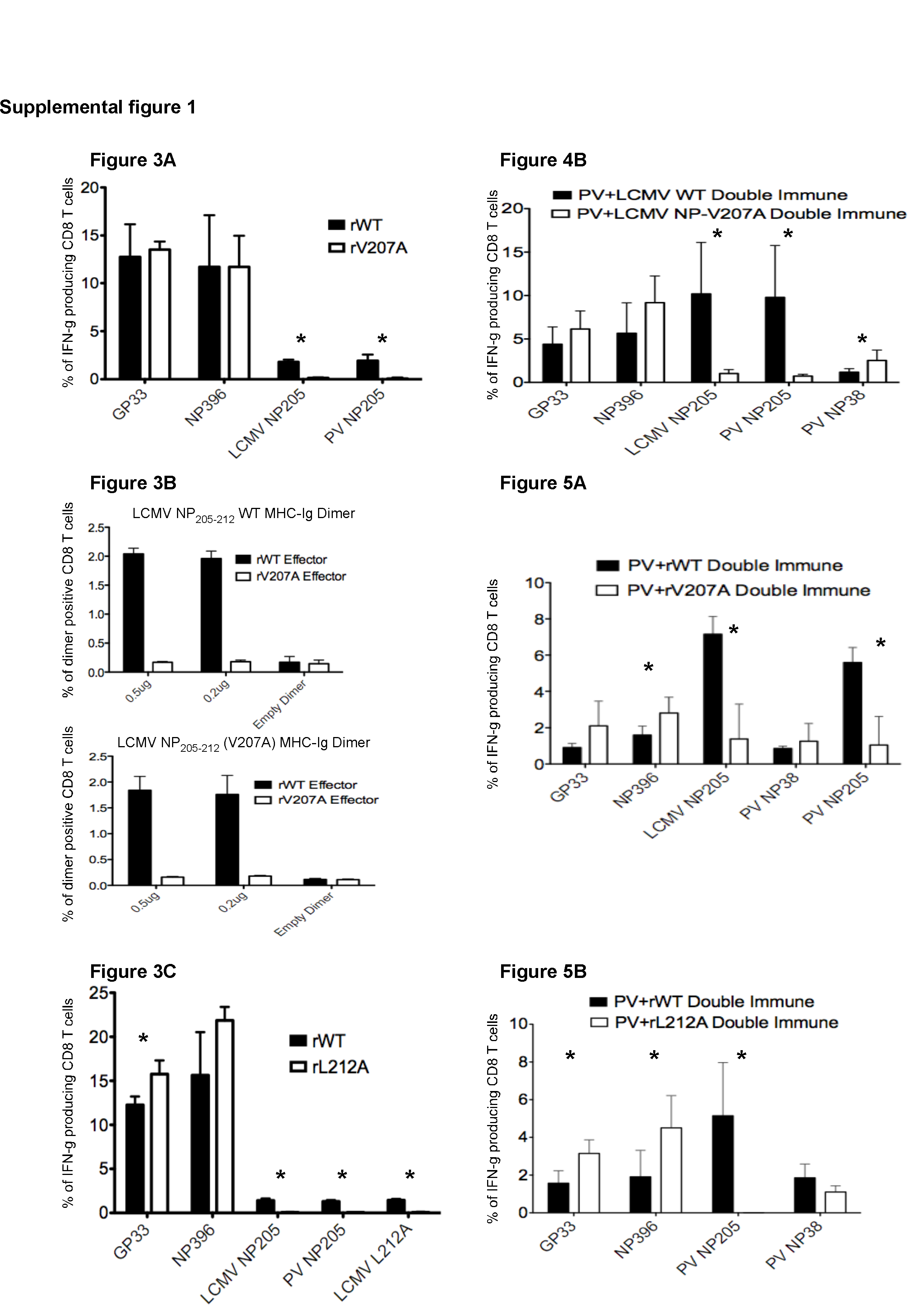

Supplement: Figure S1 — Graphic analysis of dot plots from Figures 3 , 4 , and 5 . This figure graphs the magnitude and variance of epitope-specific T cell responses from replicas associated with the representative data presented in Fig. 3A (n = 3/group), 3B (n = 2/group), 3C (n = 3/group), 4B (n = 5/group), 5A (n = 5/group), and 5B (n = 5/group). All show means ± standard deviations (p<0.05*). (TIF) [file ppat.1002633.s001.tif]
