# Supplementary material for: Loss of Anti-Viral Immunity by Infection with a Virus Encoding a Cross-Reactive Pathogenic Epitope
Source: PLoS Pathog. 2012 Apr 19;8(4):e1002633. doi: 10.1371/journal.ppat.1002633 (PMC3334890; doi:10.1371/journal.ppat.1002633)
Supplement: Table S1 — Data collection and refinement statistics. (DOC) [file ppat.1002633.s002.doc]

**Supplemental Table 1. Data collection and refinement statistics.**

| **Data Collection Statistics** | **H2Kb/NP205-LCMV** | **H2Kb/NP205-PV** | **H2Kb/NP205-LCMV-V3A** |
| --- | --- | --- | --- |
| Temperature | 100K | 100K | 100K |
| Space group | *P2_1_* | *P2_1_* | *P2_1_* |
| Cell Dimensions (a,b,c) (Å) | 66.73, 90.92, 89.05  β=111.08° | 66.19, 90.60, 89.16  β=111.11° | 66.75, 85.05, 89.19  β = 111.33° |
| Resolution (Å) | 100 - 2.50 (2.60-2.50) | 100 - 2.50 (2.60-2.50) | 100-2.30 (2.40-2.30) |
| Total number of observations | 87280 (9662) | 59208 (6509) | 299713 (33429) |
| Number of unique observations | 34537 (3711) | 34167 (3501) | 39608 (4403) |
| Multiplicity | 2.5 (2.6) | 1.7 (1.8) | 7.5 (7.6) |
| Data completeness (%) | 95.0 (97.6) | 93.0 (93.1) | 95.6 (89.6) |
| I/σ_I_ | 10.80 (4.40) | 11.12 (3.79) | 18.69 (4.61) |
| R_merge_^a^ (%) | 8.2 (23.1) | 6.6 (21.6) | 9.2 (45.6) |
| **Refinement Statistics** |  |  |  |
| Non-hydrogen atoms |  |  |  |
| Protein | 6390 | 6298 | 6305 |
| Water | 240 | 190 | 462 |
| Resolution (Å) | 2.50 | 2.50 | 2.30 |
| *R_factor_*^b^ (%) | 18.1 | 24.3 | 20.2 |
| *R_free_*^b^ (%) | 26.9 | 30.8 | 27.5 |
| Rms deviations from ideality |  |  |  |
| Bond lengths (Å) | 0.007 | 0.008 | 0.007 |
| Bond angles (°) | 1.119 | 1.170 | 1.102 |
| Ramachandran plot (%) |  |  |  |
| Most Favoured Region | 88.9 | 89.7 | 88.8 |
| Allowed Region | 9.7 | 8.6 | 10.0 |
| Generously allowed region | 0.9 | 1.4 | 0.9 |

^a^ R_merge_ = Σ  I_hkl_ - < I_hkl_ >  / ΣI_hkl_

^b^ R_factor_ = Σ_hkl_   F_o_  -  F_c_   / Σ_hkl_  F_o_  for all data except ≈ 5% which were used for R_free_ calculation

Values in parentheses are for the bin of highest resolution (approximate interval = 0.5 Å).
